# ﻿First Solenogastres (Mollusca, Aplacophora) from Puerto Rico: descriptions of two new species and notes on their coral hosts

**DOI:** 10.3897/zookeys.1261.164889

**Published:** 2025-11-26

**Authors:** M. Carmen Cobo, Corinna Breusing, Andrea M. Quattrini, Santiago Herrera, Ellen E. Strong

**Affiliations:** 1 Smithsonian Institution, National Museum of Natural History, Washington, DC, USA; 2 Graduate School of Oceanography, University of Rhode Island, Narragansett, RI, USA; 3 Department of Biological Sciences, Lehigh University, Bethlehem, PA, USA; 4 Lehigh Oceans Research Center, Lehigh University, Bethlehem, PA, USA

**Keywords:** Biodiversity, Caribbean Sea, ROV exploration, taxonomy

## Abstract

Solenogastres (Mollusca, Aplacophora) are a unique and understudied group of marine invertebrates of evolutionary and ecological significance. Their diversity remains underestimated due to frequent lack of identification by non-specialists, limited molecular data, and the complexity of their taxonomy. Here we present the first Solenogastres from Puerto Rico, expanding the group’s known geographic distribution in the Caribbean Sea. Using an integrative taxonomic approach, we describe two new species, *Dorymenia gummi***sp. nov.** and *Strophomenia boricua***sp. nov.**, and document their associations with coral hosts. These findings offer new insights into the ecology and biodiversity of these elusive mollusks.

## ﻿Introduction

Solenogastres (Mollusca, Aplacophora) are a small, yet distinctive lineage characterized by a vermiform body, the absence of a shell, and a reduced foot and mantle cavity. Although new Solenogastres species have been continuously described since their discovery in the 19^th^ century (Gegenbaur 1878), the diversity of the group remains underestimated (Todt 2013; Olson et al. 2025). To date, 313 species are formally recognized (MolluscaBase 2025) primarily from Antarctic and European waters, with many species described based on a single specimen. In some oceanic regions, Solenogastres diversity remains completely undocumented. Notably, only one species has been formally described from the Caribbean Sea and only five have been reported from nearby basins (Wirén 1892; Heath 1912; Felder and Camp 2009; Cobo et al. 2024b). However, numerous specimens from this region remain unidentified or undescribed in museum collections (Cobo unpubl. data).

Several challenges hinder efforts to document and describe solenogaster diversity, notably the scarcity of molecular data which limits robust phylogenetic analyses and accurate species delimitation (Kocot et al. 2019; Yap-Chiongco et al. 2024). Furthermore, accurate identification relies heavily on time-intensive anatomical methods, as external morphology offers limited taxonomic resolution. Although external features such as body size (0.5–300 mm), color, and the appearance of sclerites keels or body protrusions, exhibit considerable variation, they rarely provide diagnostic characters above the species level (Cobo et al. 2023). Most species are yellow, white, or brownish, and their sclerites require examination from multiple specimens under high magnification to be taxonomically informative (Scheltema and Schander 2000; Pedrouzo et al. 2014). These challenges are particularly acute within families of the non-monophyletic order ‘Cavibelonia’ (Kocot et al. 2019; Yap-Chiongco et al. 2024). Several lineages across Cavibelonia (e.g., families Proneomeniidae, Strophomeniidae, or Epimeniidae) are characterized by large body sizes, and hollow acicular sclerites. Yet, significant differences in internal anatomy suggest these features are not diagnostic above the family or genus level (Cobo et al. 2023). Thus, internal characters– particularly those of the radula, digestive glands, and reproductive and sensory organs– remain central to solenogaster taxonomy (García-Álvarez and Salvini-Plawen 2007). Although DNA barcoding and sclerite morphology can assist in preliminary classification (Bergmeier et al. 2016), species-level resolution still depends heavily on expert internal anatomical study (Bergmeier et al. 2017, 2019; Cobo et al. 2023; Olson et al. 2025). Emerging imaging techniques, such as micro-computed tomography (micro-CT), offer promising non-destructive visualization of internal anatomy (Martínez-Sanjuán et al. 2022). However, they currently lack sufficient resolution to capture crucial details of the radulae or digestive glands. As a result, histological techniques, despite being labor-intensive, remain indispensable. This methodological bottleneck exemplifies the broader ‘taxonomic impediment’ hampering the pace of solenogaster discovery.

In addition, Solenogasters are frequently overlooked, particularly by non-specialists, because of their relatively small size, cryptic nature, and the considerable sampling challenges associated with the deep sea (Todt 2013). Most described species have been collected via dredging, making live observations exceptionally rare (e.g., Pruvot 1890; Heath 1911; Salvini-Plawen 1978; Sasaki and Saito 2005; Pedrouzo et al. 2014; Cobo et al. 2024a), and laboratory-based ecological studies are exceedingly scarce (Scheltema and Jebb 1994; Okusu 2002; Todt and Wanninger 2010). Consequently, our understanding of their ecology and reproductive biology remains limited. What little is known about their feeding biology and host interactions with corals and hydrozoans (Salvini-Plawen 1972) has been inferred from gut content analysis (e.g., Salvini-Plawen 1978; García-Álvarez et al. 2000) or from molecular sequence contamination (Okusu and Giribet 2003; Meyer et al. 2010; Bergmeier et al. 2021; Olson et al. 2025), but seldom by direct observation (e.g., Scheltema and Jebb 1994; Salvini-Plawen and Benayahu 1991; Saito and Salvini-Plawen 2010). Despite these challenges, advances in deep-sea exploration using remotely operated vehicles (ROVs) offer a non-destructive alternative to dredging and facilitate the observation of larger solenogaster specimens on coral hosts (Bo et al. 2011; Zhulay et al. 2019; Taylor et al. 2021). Notably, expeditions using ROVs have significantly increased direct observations and collections, including specimens examined in this study. Larger ‘Proneomeniidae’ species are among the most frequently observed solenogasters in ROV footage due to their size and conspicuous presence on coral hosts.

In this study, we use an integrative taxonomic approach combining morphological, molecular, and ecological data to formally describe two new solenogaster species collected from Caribbean waters off Puerto Rico. Both belong to the ‘Proneomeniidae clade’ (sensu Cobo et al. 2023). Morphological analysis (particularly of radula, foregut glands, reproductive and sensory organs) was essential for identification and formal description due to limited comparative molecular data. Each species exhibited an association with a distinct octocoral host. High-resolution ROV imagery provided valuable ecological context for these relationships by documenting exact depth distribution, position on the host, and differences in the number of individuals per colony: *Strophomenia boricua* sp. nov. occurred as multiple individuals on different branches of a *Villogorgia* colony at 389 m, whereas *Dorymenia gummi* sp. nov. was observed as a single individual on a *Sibogagorgia* colony at 1458 m. The presence of cnidocytes in the gut suggest feeding on their respective hosts.

## ﻿Materials and methods

### ﻿Material examined

**Figure 1.**
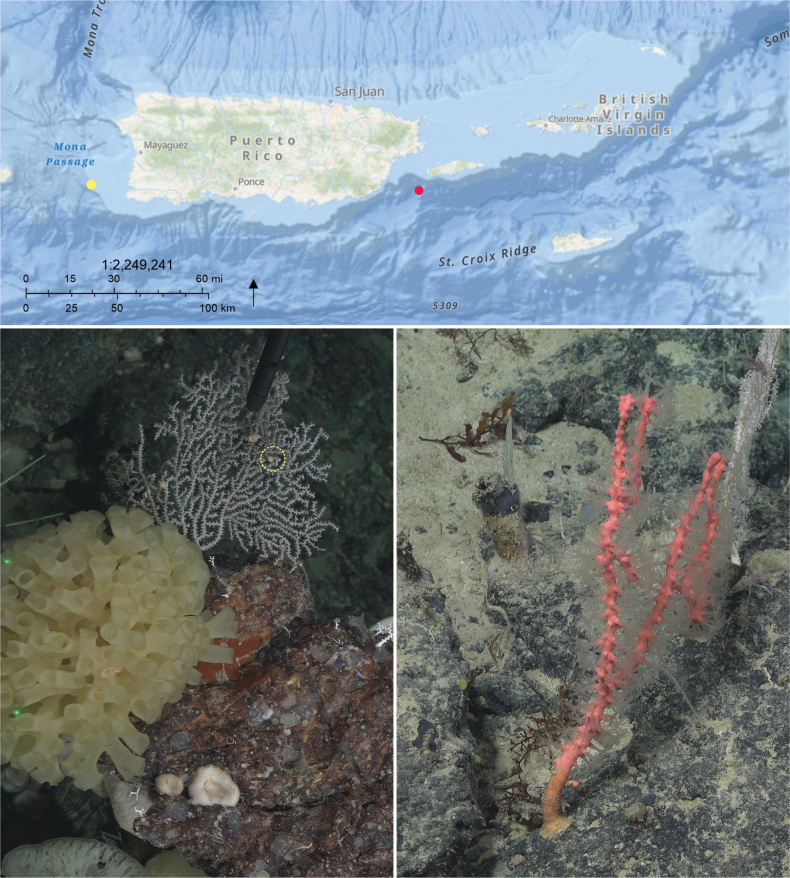
**A.** Map showing the sampling localities. Yellow dot, *Strophomenia boricua* sp. nov. (USNM 1691556; 18.034167°N, 67.407167°W; 389 m depth); red dot, *Dorymenia gummi* sp. nov. (USNM 1690452; 18.002611°N, 37.2995°W; 1458.48 m depth). **B, C.** ROV footage: **B.**Villogorgia cf. nigrescens with two individuals of *S. boricua* sp. nov. (highlighted in yellow). **C.**Sibogagorgia cf. cauliflora; *Dorymenia gummi* sp. nov. is not visible.

Three solenogaster specimens were collected from marine octocoral hosts off the coast of Puerto Rico using the ROV SuBastian during expedition FKt230417 aboard the R/V Falkor (too). Samples were collected with the ROV manipulator arm while attached to the octocoral host (Fig. 1) and placed into individual containers. Specimens were photographed alive, then preserved in 95% ethanol. Both solenogaster specimens were deposited in the Invertebrate Zoology collections of the National Museum of Natural History (USNM 1690452 and USNM 1691556) and form the basis of this study. Vouchers of their coral hosts (USNM 1689153 and USNM 1689348) were deposited in the same collection.

### ﻿Morphology

External morphology. Preserved specimens were photographed with an Olympus DSX100 or SZ51 microscope equipped with an iPhone camera mounted in a Phone Skope case. Body length (along the midline) and dorso-ventral height were measured in lateral view and compared with field images.

Sclerites. A mid-body fragment from each specimen was air-dried and imaged uncoated under low-vacuum conditions with a Zeiss EVO MA15 scanning electron microscope (SEM). The same fragments were later used for DNA extraction. Individual sclerites were dislodged using a minuten pin onto a slide with distilled water, air dried, mounted with DEPEX and studied and measured under an Olympus BX63F compound microscope.

Histology. Anterior and posterior body regions were dehydrated, embedded in paraffin, and sectioned transversely at 5 μm using a Reichert-Jung 820 II Histocut microtome. Sections were stained with Mallory’s trichrome. The protocol followed Gil-Mansilla et al. (2008) with minor modification: xylene exposure during embedding was limited to 15 min (until tissues became translucent), paraffin infiltration was reduced from three to two hours, and staining with aniline blue/orange G was reduced from 20 to 15 min. Sections were imaged with an Olympus BX63F compound microscope.

### ﻿Molecular analysis

DNA extraction and sequencing. DNA was extracted using the AutoGen 965+ platform following the manufacturer’s protocol. Extracted DNA was quantified with the ThermoFisher Scientific Quant-IT 1× dsDNA high-sensitivity assay kit on a SpectraMax ID3 microplate reader. Sequencing libraries were prepared with the NEBNext Ultra II FS DNA Library Prep Kit in half reactions, using a fragmentation time of 3 min and automatic bead cleanup on an Opentrons system. Samples were dual-indexed with iTru i5 and i7 barcodes onto y-yoke adaptors during limited cycle PCR (Glenn et al. 2019). Libraries were pooled, quality-checked with an Agilent TapeStation High Sensitivity D1000 ScreenTape assay and sequenced on a NovaSeqX platform at the Oklahoma Medical Research Foundation, targeting 20 million pair-end reads (2 × 150 bp) per sample.

### ﻿mtCOI, 18S rRNA and 28S rRNA barcode assembly

Raw reads were assessed for quality with FastQC v0.12.1 (https://www.bioinformatics.babraham.ac.uk/projects/fastqc/) and subsequently trimmed with fastp v, 0.23.4 (Chen et al. 2018) and Trimmomatic v. 0.39 (Bolger et al. 2014) to remove polyG tails, adaptors and low-quality bases. Contaminants were removed by mapping the trimmed reads against the human and PhiX genomes using Bowtie2 v. 2.5.3 (Langmead and Salzberg 2012). Clean reads were aligned against the BOLD, SILVA SSU and SILVA LSU databases with bbmap v. 39.06 (https://sourceforge.net/projects/bbmap/) and matching reads were assembled with metaSPAdes v. 3.15.5 (Nurk et al. 2017) using default settings. To identify rRNA genes, we further ran barrnap v. 0.9 (https://github.com/tseemann/barrnap) on metagenomic assemblies for each sample. The taxonomic identity of the resulting mtCOI, 18S rRNA and 28S rRNA barcodes was determined through BlastN searches (Camacho et al. 2009) and the longest sequence for each barcode and target organism was extracted using a combination of bedtools v. 2.31.1 (Quinlan and Hall 2010), seqtk v. 1.4 (https://github.com/lh3/seqtk) and seqkit v. 2.8.1 (Shen et al. 2016). To improve mitochondrial barcode recovery, we also assembled partial mitochondrial genomes with MitoFinder v. 1.4.1 (Allio et al. 2020) and MITObim v. 1.9.1 (Hahn et al. 2013) using the initial COI assemblies as seeds (*Dorymenia gummi* sp. nov. 4,456 bp; *Strophomenia boricua* sp. nov.: 7,153 bp). Full-length COI barcode sequences were extracted from these assemblies and used for further analysis. All the newly generated sequences are deposited in GenBank under Bioproject PRJNA1338364.

### ﻿Phylogenetic analysis

COI sequences were retrieved from GenBank (Table 1) to match taxon sampling from Cobo et al. 2023. Sequences were aligned using MAFFT v. 7.487 (https://www.ebi.ac.uk/Tools/msa/mafft/; Katoh and Standley 2013) and manually inspected and edited in Geneious Prime to ensure no gaps or frameshifts were present.Phylogenetic analyses were performed in IQ-TREE 2 (Minh et al. 2020) under a maximum-likelihood framework. The best-fitting nucleotide substitution model was selected using ModelFinder (Kalyaanamoorthy et al. 2017), and node support was assessed with 1,000 ultrafast bootstrap replicates (Hoang et al. 2018).

**Table 1.** Species included in the phylogenetic analysis of partial COI sequences (taxon selection from Cobo et al. 2023). Voucher numbers: (ALMNH) Alabama Museum of Natural History, Tuscaloosa, AL, USA; (AP) Reference number of vouchers from Kocot et al. (2019); (BioSample) From De Oliveira et al. (2016); (MNHN) Muséum national d’Histoire naturelle, Paris, France; (MCZ) Museum of Comparative Zoology, Harvard University, Cambridge, MA, USA; (SAMN) (USNM) Smithsonian National Museum of Natural History; (ZMBN) Museum of Zoology at the University of Bergen, Norway; (ZSM) Zoologische Staatssammlung München (the Bavarian State Collection of Zoology), Munich, Germany.

Species ID	Voucher number	GenBank number	Reference
*Proneomenia franziae*	MNHN-IM-2013-66993	OQ600029	Cobo et al. 2023
*Proneomenia occulta*	MNHN-IM-2013-66992	OQ600028	Cobo et al. 2023
*Proneomenia satiata*	MNHN-IM-2013-61611	OQ600026	Cobo et al. 2023
*Proneomenia* sp.	ALMNH:Inv:24165	OQ600023	Cobo et al. 2023
*Proneomenia* sp.	ALMNH:Inv:24163	OQ600021	Cobo et al. 2023
*Proneomenia gerlachei*	ALMNH:Inv:24161	OQ600019	Cobo et al. 2023
*Proneomenia sluiteri*	ZMBN 94113	KJ568517.1	Kocot and Todt 2014
*Proneomenia custodiens*	ZMBN 94109	KJ568518.1	Kocot and Todt 2014
*Dorymenia boucheti*	MNHN-IM-2013-50092	OQ600025	Cobo et al. 2023
*Dorymenia sarsii*	n/a	OQ600024	Cobo et al. 2023
*Dorymenia tricarinata*	Ap231.5R	OQ600547	Cobo et al. 2023
*Dorymenia* sp.	ALMNH:Inv:24159	OQ600017	Cobo et al. 2023
*Dorymenia* sp.	ALMNH:Inv:24160	OQ600018	Cobo et al. 2023
*Dorymenia* sp.	ALMNH:Inv:24162	OQ600020	Cobo et al. 2023
*Dorymenia gummi* sp. nov.	USNM 1690452		Present work
*Kruppomenia genslerae*	ZSM Mol 20170348	MN531184.1	Ostermair et al. 2018
*Simrothiella margaritacea*	Ap189.1R	OQ600548	Cobo et al. 2023
*Unciherpia hirsuta*	MNHN-IM-2019-18279	OQ600031	Cobo et al. 2023
*Hypomenia sanjuanensis*	Ap183.1R	OQ600549	Cobo et al. 2023
*Epimenia babai*	MCZ DNA100843	AY377724.1	Okusu and Giribert 2003
*Epimenia autralis*	MCZ DNA100841	AY377722.1	Okusu and Giribert 2003
*Anamenia gorgonophila*	MNHN-IM-2019-18270	OQ600030	Cobo et al. 2023
*Strophomenia boricua* sp. nov.	USNM 1691556		Present work
*Wirenia argentea*	n/a	MG855759.1	Mikkelsen et al. 2018
*Gymnomenia pellucida*	BioSample:SAMN06141848	OQ600550	Cobo et al. 2023

### ﻿Identification of coral hosts

Two octocoral hosts (Anthozoa: Octocorallia) were identified using genomic data and morphological characters. Octocoral hosts were preliminarily identified by examining skeletal axis types and sclerite morphology following Bayer et al. (1983), resulting in tentative identifications of two species: Sibogagorgia cf. cauliflora Herrera, Baco & Sánchez, 2010 (USNM 1689348) and *Villogorgia nigrescens* Duchassing & Michelotti, 1860 (USNM 1689153). Raw data from each coral host were treated as above, and trimmed reads were subsequently assembled with MitoFinder v. 1.4.1 using a reference octocoral database downloaded from GenBank (Quattrini et al. 2023). A mitogenome for the *V. nigrescens* schizotype (USNM 1440187) was also assembled for taxonomic comparison. Assembled mitogenomes were visually inspected, manually curated, and circularized (if necessary) in Geneious Prime.

Sequences for the mtMutS gene, a mitochondrial, informative DNA barcode for octocorals (McFadden et al. 2011), were then extracted from the assemblies. The *Sibogagorgia* sequence was aligned with Muscle in AliView v. 1.26 (Larsson 2014) against *S. cauliflora* type data from GenBank (holotype: GQ293317, USNM 112229; paratypes: GQ293310, USNM 112230; KP7USNM54831). The *Villogorgia* sequence was aligned with the mtMutS data from the *V. nigrescens* schizotype. mtMutS sequences were also blasted against the nr/nt collection in GenBank. Newly acquired mtMutS data is on GenBank under BioProject PRJNA1338364.

## ﻿Results

The two newly described Solenogastres species belong to distinct families within the order ‘Cavibelonia’ Salvini-Plawen, 1978, which has been shown to be non-monophyletic (Kocot et al. 2019; Yap-Chiongco et al. 2024). One is placed in Proneomeniidae, a family identified as polyphyletic in a recent study (Cobo et al. 2023), and the other in Strophomeniidae, which, with Epimeniidae, was found to be nested within Proneomeniidae (Cobo et al. 2023; Yap-Chiongco et al. 2024). Nevertheless, our phylogenetic analyses recover Proneomeniidae as monophyletic, with Strophomeniidae and Epimeniidae as its sister groups. Given the taxonomic uncertainty, we follow the currently accepted classification outlined in García-Álvarez and Salvini-Plawen (2007), pending further revision.

### ﻿Systematics

**Order Cavibelonia Salvini-Plawen, 1978**

**Family Proneomeniidae Mitchell, 1892**

#### ﻿Genus Dorymenia

Taxon classificationAnimaliaCavibeloniaProneomeniidae

Heath, 1911

A6AD5D8F-AC72-5D32-8562-E5E581EB2FF4

##### Type species.

*Dorymenia acuta* Heath, 1911, by original designation. Type locality. Santa Barbara Islands (California, USA) (Albatross St. 4415); 550–1150 m depth.

##### Other included species.

*D. acutidentata* Salvini-Plawen, 1978; *D. ancora* McCutcheon, Kocot & Cobo, 2022; *D. antarctica* (Thiele, 1913); *D. boucheti* Cobo & Kocot, 2023; *D. cristata* Salvini-Plawen, 1978; *D. discoveryi* (Nierstrasz, 1908); *D. harpagata* Salvini-Plawen, 1978; *D. hesperidesi* García-Álvarez, Urgorri & Salvini-Plawen, 2000; *D. hoffmani* Salvini-Plawen, 1978; *D. interposita* Salvini-Plawen, 1978; *D. longa* (Nierstrasz, 1902); *D. lucida* McCutcheon, Kocot & Cobo, 2022; *D. menchuescribanae* García-Álvarez, Urgorri & Salvini-Plawen, 2000; *D. parvidentata* García-Álvarez & Urgorri, 2003; *D. paucidentata* Salvini-Plawen, 1978; *D. peroneopsis* Heath, 1918; *D. profunda* Salvini-Plawen, 1978; *D. quincarinata* (Ponder, 1970); *D. sarsii* (Koren & Danielssen, 1877); *D. singulatidentata* Salvini-Plawen, 1978; *D. tanifa* McCutcheon, Kocot & Cobo, 2022; *D. tetradoryata* Salvini-Plawen, 1978; *D. tricarinata* (Thiele, 1913); *D. troncosoi* García-Álvarez, Urgorri & Salvini-Plawen, 1998; *D. usarpi* Salvini-Plawen, 1978; *D. vagans* (Kowalevsky & Marion, 1887); *D. weberi* (Nierstrasz, 1902).

#### ﻿ Dorymenia gummi

Taxon classificationAnimaliaCavibeloniaProneomeniidae

Cobo & Strong sp. nov.

8ABC0379-6479-5C96-9418-BB08D97B6904

https://zoobank.org/FA9A9953-5B1F-418F-9120-430312F2A99C

Figs 2, 3

##### Type material.

***Holotype***: USNM 1690452, East of Puerto Rico, Caribbean Sea, 18.002611°N, 37.2995°W; 1458.48 m depth. Serial sections (34 slides; 5 µm), a mid-body fragment preserved in 95% ethanol, and DNA barcode sequences (mtCOI, 18S rRNA, 28S rRNA).

##### Diagnosis.

Animal slender, elongate (18 × 1–1.8 mm) without posterior digitiform projection. Sienna in color in life. Cuticle thick, hollow acicular sclerites dominate. Pedal groove with knife-shaped scales. Polystichous radula with up to 100 uniform elongate teeth per row. A pair of single copulatory stylets, rounded in cross-section. With abdominal spicules. Without seminal receptacles. With a dorsoterminal sensory organ.

##### Description.

**Figure 2.**
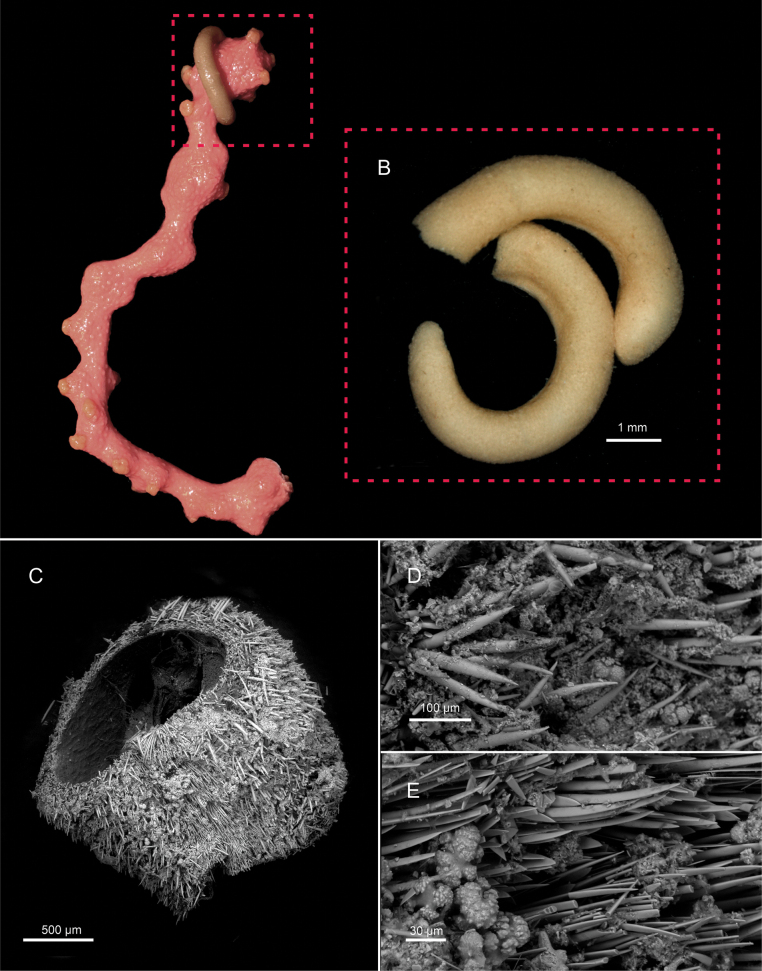
External characters of *Dorymenia gummi* sp. nov. (holotype USNM 1690452). **A.** Live specimen on the coral host; **B.** Specimen after preservation in ethanol; **C.**SEM image of the mid-body region; **D.** Acicular sclerites in the dorsal region of the body; **E.** Sclerites of the ventral region of the body.

***External aspect and sclerites*.** Elongated animal (18 mm long, 1–1.8 mm wide). Sienna in color in life (Fig. 2A), cream-white when preserved in ethanol (Fig. 2B). The anterior region of the body is broader than the posterior, which is slightly more tapered but lacks a digitiform projection (Fig. 2B). Sclerites do not protrude externally, and their arrangement is not visible without magnification. Pedal groove and atrio-buccal cavity externally distinguishable. The predominant sclerite type is hollow acicular, spines arranged in several layers in all the surface of the body but less abundant in the ventral region (Fig. 2C, D). Two main morphotypes are observed: 1. Slightly curved with a rounded distal end and an internal cavity extending along most of the length (160–200 μm long, 10–15 μm wide); 2. Straight with a pointed distal end and an internal cavity running along the entire length (140–180 μm long, 8–12 μm wide). Along the pedal groove (Fig. 2C, E), acicular sclerites are sparse, replaced by long flat solid sclerites (160–190 μm long, 15–20 μm wide) and characteristic knife-shaped scales (80–90 μm long, 20–22 μm wide). Paddle-shaped scales lacking.

**Figure 3.**
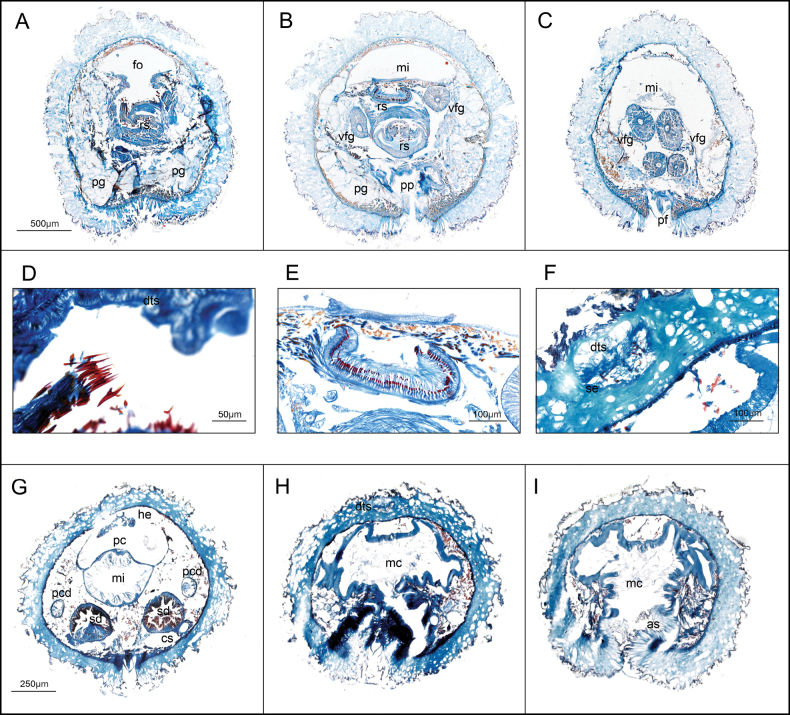
Histological sections of *Dorymenia gummi* sp. nov. (holotype USNM 1690452). **A.** Anterior region of the radula apparatus; **B.** Mid region of the radula apparatus, with well-developed ventrolateral foregut glands; **C.** Posterior folded region of the ventrolateral foregut glands; **D.** Detail of the teeth; **E.** Detail of a radula row (94 teeth); **F.** Detail of the dorsoterminal sensory organ; **G.** Posterior region of the paired spawning duct; **H.** Mantle cavity (closure) with the dorsoterminal sensory organ; **I.** Mantle cavity (opening) with abdominal spicules. Abbreviations: as – abdominal spicules, dts – dorsoterminal sensory organ, fo – foregut, he – heart, mc – mantle cavity, mi – midgut, pc – pericardium, pcd – pericardioducts, pf – pedal fold, pg – pedal gland, pp – pedal pit, rs – radular sac, vfg – ventrolateral foregut glands.

***Internal anatomy*.** Cuticle thick (90–200 μm) with simple epidermal papillae, whose distal portions are most evident in the outermost layer, while intermediate parts are less distinct in serial sections. Anterior follicular pedal glands forming a prominent glandular mass that surrounds the anterior foregut (Fig. 3A, B). Pedal pit posterior, located in the radular region (350 μm length, 130–340 μm width, 30–130 μm height) (Fig. 3B). The pedal groove contains a single triangular fold (80–100 × 20–30 μm) (Fig. 3C). Cerebral ganglion, nearly circular in cross-section (180 μm length, 100–200 μm width, 80–180 μm height). Atrium (360 μm long, 250–500 μm wide, 80–250 μm high) with numerous single papillae, with four larger ones: two attached dorsally and two ventrally, flanking the atrial opening. These larger papillae are interpreted as the atrial sense organs. The mouth opens dorsally at the posterior end of the atrium and continues into a rounded, tubular foregut which continues almost parallel to the pedal groove. In the radular region, the foregut enlarges and is mushroom shaped in cross section (Fig. 3A). Radular apparatus formed by a polystichous radula, a voluminous radular sac (320 μm long, 100–120 μm wide, 50–80 μm high) and a subradular pouch of similar dimension (Fig. 3A, B). Each radular row (Fig. 3E) comprises 94–102 uniform, elongate teeth (7–9.4 μm long, 1.5–3 μm wide) with a straight base, a small lateral denticle, and a longer, uncurved principal denticle (Fig. 3D). Ventrolateral foregut glands are of type C (García-Álvarez and Salvini-Plawen 2007) (*Epimenia*-type: Handl and Todt 2005), consisting of long, straight tubes that connect to the foregut via the radular sac and are folded in the post-radular region where four tubes are visible in section (Fig. 3C). A dorsal caecum is absent, and the midgut does not show distinct serial constrictions. The gonoducts are not clearly developed. The pericardium is broad and contains a rounded heart attached to its dorsal wall, with reproductive cells (Fig. 3G), but in the most posterior part it is straight and paired. The pericardioducts (810 μm in length, 80–95 μm in diameter) connect with the pericardium in its paired region and with the spawning duct at its origin. The spawning duct originates as two independent tubes (640 μm length, 80–100 μm in diameter) that fuse into a single duct in its mid-posterior region (450 μm length, 70–140 μm in diameter) and opens dorsally at the ciliated posterior end of the mantle cavity. The ventral walls of the mantle cavity are flanked by abdominal spicules (Fig. 3I). The mantle cavity is large (640 μm length, opening about 400 μm long) and features dorsal ear-like protrusions (Fig. 3I). A pair of single, rounded copulatory spicules is present (up to 1 mm long, 50–60 μm in diameter). A rounded dorsoterminal sensory organ is located dorsally, just above the closure of the mantle cavity (Fig. 3F, H).

##### Etymology.

-*gummi* Latin noun (alternative form of *cummis*) meaning “gum,” referring both to the soft, elongated body of the solenogaster, reminiscent of a “gummy worm”, and to its ecological association with the so-called “bubblegum coral” (*Paragorgia* sp.). Used as a noun in apposition.

##### Remarks.

This species is placed in genus *Dorymenia* (Proneomeniidae) based on the presence of copulatory stylets (Heath 1918; Scheltema and Schander 2000; García-Álvarez and Salvini-Plawen 2007; García-Álvarez et al. 2009). It differs from other congeners by the exceptionally high number of radular denticles and absence of a digitiform posterior projection. The radula of *Dorymenia gummi* sp. nov. bears approximately 100 denticles, a distinguishing feature within the genus (McCutcheon et al. 2022: table 2). Among the described species, only *D. hesperedesi* from Antarctica has a similar radular count (100 denticles), but *D. gummi* sp. nov. differs from this species in key internal characters (presence of a single dorsoterminal sensory organ vs three and lacks seminal receptacles) and occurs at greater depths (>1000 m vs 235 m). The new species also differs externally from the geographically proximate *D. peroneopsis* by lacking a prominent digitiform projection. This new solenogaster species is associated with a coral host, Sibogagorgia cf. cauliflora. Only one specimen was observed on the colony, ~ 22 cm in height.

**Table 2.** Main distinguishing characters of *Strophomenia* species. Coral host identifications follow the names provided in the literature. The number of examined specimens (*n*) is indicated below each species name, based on the respective original description; + indicates present; - not available/ known.

	*S. lacazei* (*n* = 3)	*S. debilis* (*n* = 1)	*S. indica* (*n* = 28)	*S. ophidiana* (*n* = 1)	*S. regularis* (*n* = 1)	*S. scandens* (*n* = 3)	*S. boricua* (*n* = 1)
**Distribution**	La Calle (Algeria)	Buton Strait (Indonesia)	Kei Islands, Java, Bay of Bima, W Salawati, Timor (Indonesia)	Honshu Islands (Japan)	Honshu Islands (Japan)	Bird Islands (Hawaii)	Puerto Rico
**Depth (m)**	Littoral	75–94	18–506	95–140	130–180	625–1035	389
**Size (mm)**	45 × 2–3	6 × 0.75	11–39 × 1–2.5	43 × 2.5	9 × 1	32–39 × 1.6–2.1	18 × 1–1.8
**Habitus**	Pale brown	Brown	Brown to yellow	Creamy white	?	?	Brown to orange
**Pedal fold**	1 triangular	3 triangular equal size	1 medial and two small lateral	?	1 small	1 medial and 2 small lateral	1 almost rectangular
**Ventrolateral foregut glands**	Fusion with foregut not observed. Only right side. Folded.	Fusion with foregut ventral as separated tubes. Folded.	Paired in all their extension.	Fusion with foregut ventral.	?	Fusion with foregut ventral single (ventral?). Then continue as 2 tubes.	Fusion with foregut dorsal and single. Two tubes, first only on the right side. Slightly folded.
**Genital opening**	Paired	Paired	Paired	Paired	Paired	Single (short)	Single
**Radular sac**	-	Rudimentary	-	-	?	-	Rudimentary
**Dorsoterminal sensory organ**	Not observed	-	+	+	+	+	+
**Seminal receptacle**	8	?	≥ 13	23	12	15–18	5
**Coral host**	’Muricea’	’Gorgonids’	’Gorgonids’	‘*Acanthogorgia angustiflora*’	‘Dendronephyta’	‘*Acanthogorgia armata*’	*Villogorgia nigrescens*
**Reference**	Pruvot 1899: figs 23–31	Nierstrasz 1902: pl. IV figs 114–117	Nierstrasz 1902: pl. II figs 101–112	Heath 1911: pls 1, 8, 9, 18	Heath 1911: pls 24, 26	Heath 1911: pls 6, 12, 13, 32, 37	This work

### ﻿Family Strophomeniidae Salvini-Plawen, 1978

#### ﻿Genus Strophomenia

Taxon classificationAnimaliaCavibeloniaProneomeniidae

Pruvot, 1899

5B861B1E-EDF4-54EE-BA3B-859DF1A879B2

##### Type species.

*Strophomenia lacazei* Pruvot, 1899, by monotypy. **Type locality.** La Calle (Algeria), Mediterranean Sea; littoral.

##### Other included species.

*S. debilis* (Nierstrasz, 1902); *S. indica* (Nierstrasz, 1902); *S. regularis* Heath, 1911; *S. ophidiana* Heath, 1911; *S. regularis* Heath, 1911; *S. scandens* (Heath, 1905).

#### ﻿ Strophomenia boricua

Taxon classificationAnimaliaCavibeloniaProneomeniidae

Cobo & Strong sp. nov.

13D14C15-53AC-579C-8975-08E748AA6C77

https://zoobank.org/FA9A9953-5B1F-418F-9120-430312F2A99C

Figs 4, 5

##### Type material.

***Holotype***: USNM 1691556, West Puerto Rico, Caribbean Sea, off Cabo Rojo, Mona Passage; 18.034167°N, 67.407167°W; 389 m depth. Serial sections (28 slides 5 µm), fragment of specimen (mid-body region) in 95% ethanol, DNA sequences (mtCOI, 18S rRNA, 28S rRNA).

##### Diagnosis.

Animal slender, elongate (18 × 1–1.8 mm), sienna to orange in color in life. Cuticle thick, hollow acicular sclerites dominate. Epidermal papillae pedunculate. Radula lacking; radular sac vestigial. Ventrolateral foregut glands of type B, fusing dorsally in the foregut. Tubes running only on the right side of the body in their anterior region. Five bundled seminal receptacles on each side. Dorsoterminal sensory organ well developed. Spawning ducts paired. Respiratory folds, copulatory stylets, and abdominal spicules lacking.

##### Description.

**Figure 4.**
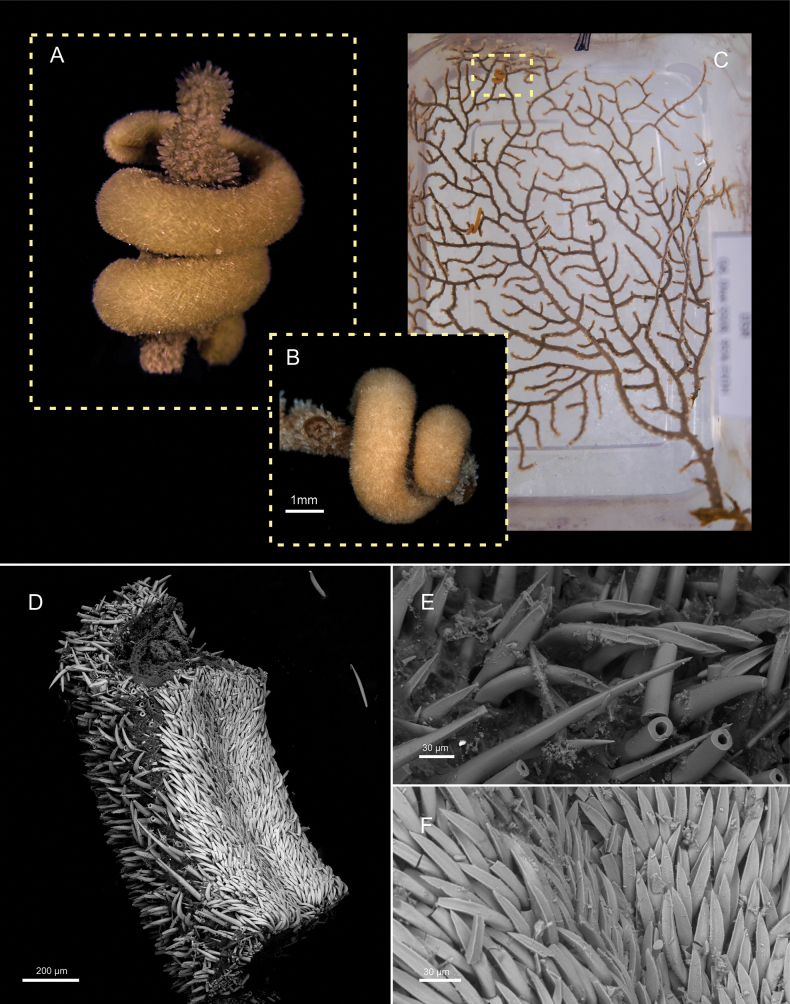
External characters of *Strophomenia boricua* sp. nov. (holotype USNM 1691556). **A.** Live specimen on the coral host; **B.** Specimen after preservation in ethanol; **C.** Field image of the specimens on the coral host. The specimen studied in this work is marked in the image; **D.**SEM image of the mid-body region; **E.** Acicular sclerites in the dorsal region of the body; **F.** Sclerites of the ventral region of the body.

***External aspect and sclerites*.** Animal slender, elongate (18 mm long, 1–1.8 mm wide) with rounded ends (Fig. 4A, B). In life, the body is brown to orange (Fig. 4C); it becomes dark brown when preserved in ethanol (Fig. 4A, B). Sclerites do not protrude externally, and their arrangement is not visible without magnification. With three types of hollow acicular sclerites located in the dorsal and mid body region (Fig. 4D, E): 1) the main type (140–200 μm long, 15–20 μm wide) is strongly curved, with a characteristic distal end bearing three ridges; 2) the second most abundant type (140–160 μm long, 15–20 μm wide) has also ridged distal ends but emerges straight from the body surface; 3) less common are slightly curved hollow acicular sclerites with a pointed distal end and a rounded base (120–200 μm long, 15–30 μm wide). Along the pedal groove, the arrangement of sclerites is dense and distinctive (Fig. 4D, F). Acicular sclerites like those on the dorsal surface are found, also bearing ridged distal ends, but with a flatter tip and curvature oriented in the opposite direction (120–140 μm long, 12–15 μm wide). In the most ventral portion, these diminish in number and are replaced by numerous knife-shaped scales, characteristic of the pedal groove (40–60 μm long, 10–18 μm wide). The pedal groove and atrio-buccal cavity (Fig. 4A) are externally distinguishable.

**Figure 5.**
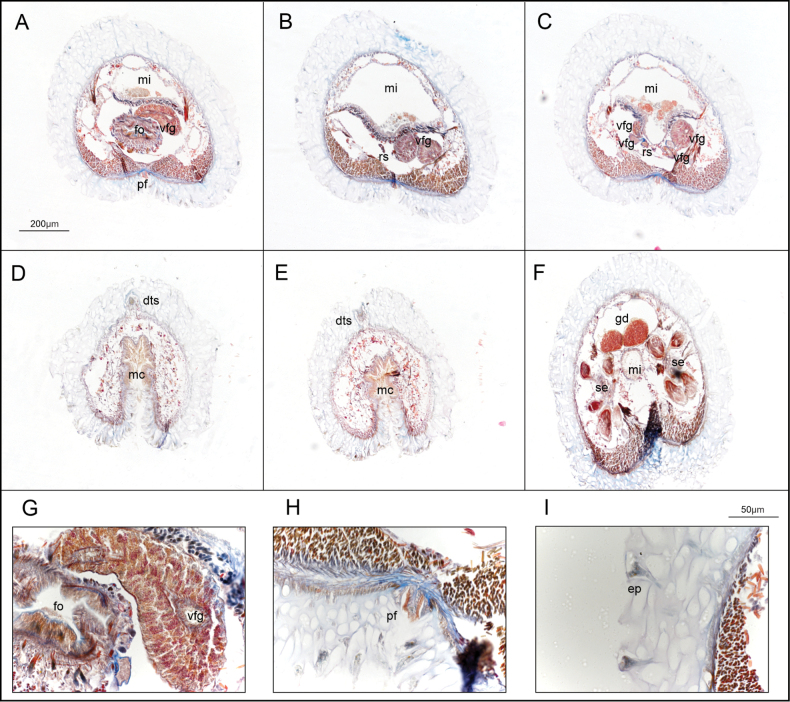
Histological sections of *Strophomenia boricua* sp. nov. (holotype USNM 1691556). **A.** Dorsal connection of the ventrolateral foregut glands with the foregut; **B.** Ventrolateral foregut glands located on the right side of the body; **C.** Ventrolateral foregut glands at both sides of the body and vestigial radular sac; **D, E.** Folded mantle cavity and the dorsoterminal sensory organ; **F.** Seminal receptacles in bundles; **G.** Detail of the ventrolateral foregut glands (corresponds with **A.**); **H.** Detail of the pedal fold and the cuticle; **I.** Detail of the cuticle with the epidermal papillae. Abbreviations; dts – dorsoterminal sensory organ, ep – epidermal papillae, fo – foregut; gd – gonad, mc – mantle cavity, mi – midgut, pf – pedal fold, rs – radular sac, vfg – ventrolateral foregut glands.

***Internal anatomy*.** Cuticle thick with sclerites in several layers (Fig. 5). The thickness of the cuticle is constant through the body, but thinner ventrally (up to 200 μm dorsal, 50–80 μm ventral) and is traversed by abundant pedunculate epidermal papillae (Fig. 5I). Pedal groove contains a single, almost rectangular, pedal fold (25 × 12.5 μm) (Fig. 5H). The atrium (250 μm long, 200–210 μm wide, 90–100 μm high) opens ventrally, with small papillae anteriorly and two main sensory structures ventrally. Cerebral ganglia almost rectangular in cross-section (115 μm long, 100 μm wide, 90 μm high). Pedal pit densely ciliated, triangular in shape (110 μm long, 60 μm wide, 30 μm high). The mouth opens at the posterior end of the atrium and continues into a robust, muscular foregut, almost circular in cross section (525 μm long, 80–120 μm wide, 60–100 μm high). A dorsal caecum extends to the mid-anterior region of the body. The foregut merges directly with the midgut caecum in the radular region, without forming a distinct esophagus. Ventrolateral foregut glands are of type B (García-Álvarez and Salvini-Plawen 2007) (Fig. 5G) and connect dorsally to form a single duct in the foregut (Fig. 5A). The foregut glands continue first as two independent tubes only on the right side of the midgut (Fig. 5B) and posteriorly they are positioned on both sides of the midgut and are folded: in some sections, each side shows a main tube and one or two smaller ventral ones (Fig. 5C). A rudimentary radular sac is present after the fusion of the foregut and the dorsal caecum, but no radula is developed (Fig. 5C). Midgut with serial constrictions. Spawning ducts are paired along most of their length (450 μm length, 80–100 μm width, 50–60 μm heigh), open ventrally into the mantle cavity (120 μm length, 20–30 μm width, 20–40 μm heigh). Mantle cavity small, ciliated (520 μm length, 40–50 μm width, 120–140 μm heigh) (Fig. 5D, E). Bundles of five seminal receptacles on each side, located in the connection of the pericardioducts with the anterior portion of the spawning ducts (Fig. 5F). A single, well-developed dorsoterminal sensory organ is present (Fig. 5D, E). Respiratory folds, copulatory stylets, and abdominal spicules are absent.

##### Etymology.

‘Boricua’ is a term that refers to a native of Puerto Rico or someone of Puerto Rican descent. It originates from ‘*Borikén*’ (also spelled ‘*Borinquen*’), the Taíno name for the island of Puerto Rico, and is widely used by Puerto Ricans to refer to themselves. The use of *boricua* aims to honor the cultural identity of Puerto Rico and highlights the geographic origin of the species. Used as a noun in apposition.

##### Remarks.

**Figure 6.**
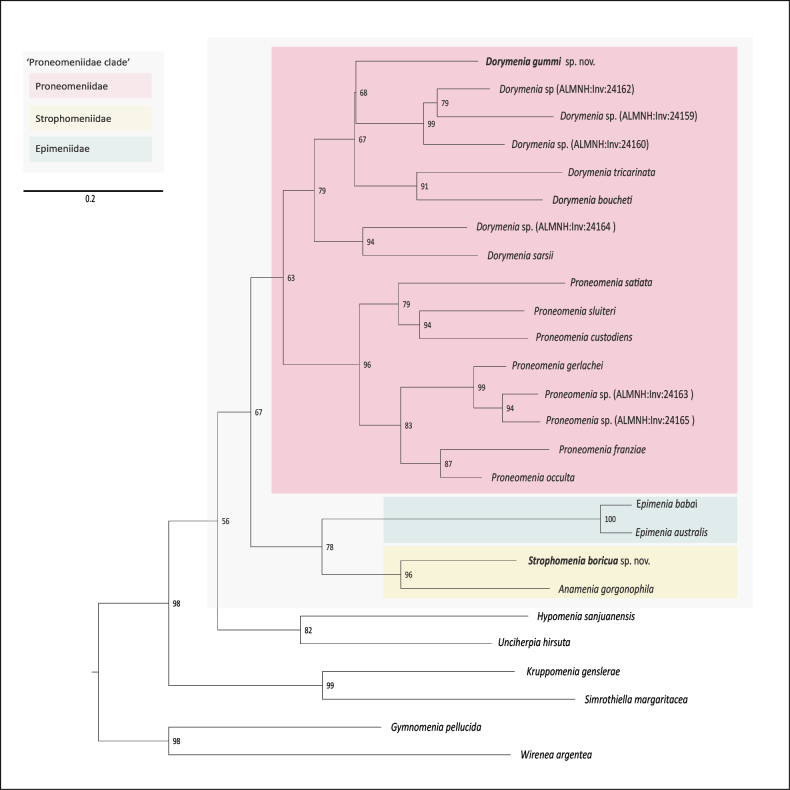
Maximum likelihood phylogenetic tree based on COI showing the position of *Dorymenia gummi* sp. nov. (USNM 1690452) and *Strophomenia boricua* sp. nov. (USNM 1691556). Ultrafast bootstrap support values are shown. Families of interest are indicated by colors. Dataset selected based on Cobo et al. (2023).

This species is assigned to genus *Strophomenia* (Strophomeniidae) based on the absence of a radula, and the presence of type B ventrolateral foregut glands and of bundled seminal receptacles (García-Álvarez and Salvini-Plawen 2007). It is distinguished from its congeners in the combination of reproductive features (fewer seminal receptacles) and arrangement of foregut glands (Table 2). Molecularly, it is recovered as sister to *Anamenia gorgonophila* (Kowalevsky, 1880) (Fig. 6). *Strophomenia boricua* sp. nov. is distinguished from all described species of the genus (Table 2) by a unique combination of internal features, including a reduced number of seminal receptacles (five per side vs. 8–23 in other species) and a rudimentary radular sac (previously only reported in *S. debilis*; Nierstrasz 1902). It further differs from *S. debilis* in the asymmetrical arrangement of the ventrolateral foregut glands and the nearly rectangular shape of the pedal fold (triangular in all other known species). The discharge of the paired spawning ducts as a single short tube in the mantle cavity, shared only with *S. scandens* (Heath 1905), also supports its distinction. The genus *Strophomenia* is well supported based on the current morphological diagnostic characters: seminal receptacles, absence of a radula, distinctive pharyngeal gland structures (type B and commonly located on just one side of the body after the connection with the foregut), a well-developed dorso-terminal sensory organ, and paired genital openings (except for *S. scandens*, although the single opening is very short; Heath 1911). A more detailed analysis of the sclerites may also reveal that the carinated distal ends observed in *S. boricua* sp. nov. as well as the arrangement of the sclerites in the pedal region (observed for other *Strophomenia* species; Heath 1911) are also diagnostic of the genus. However, distinguishing species within *Strophomenia* remains challenging. Definitive diagnostic characters are limited, and the distinction is based on a combination of characters that must be studied in detail (Table 2). Ecological traits, particularly coral-host associations, may offer valuable clues for species delimitation within the genus. This new solenogaster species is associated with the coral host *Villogorgia nigrescens*. Two specimens were observed on a colony, ~ 30 cm in height.

### ﻿DNA barcoding and phylogenetic analysis

Full-length COI sequences as well as partial 18S and 28S rRNA barcodes were obtained for the newly described species (GenBankBioProject = PRJNA1338364). The solenogaster COI sequences were used to confirm morphological identifications through a phylogenetic analysis based on publicly available sequences (Table 1, Fig. 6) and following Cobo et al. (2023). *Dorymenia gummi* sp. nov. was recovered as the sister taxon of a clade including three other *Dorymenia* species (bs = 68) and nested within a clade comprising all included species of the genus (bs = 79). *Strophomenia boricua* sp. nov. was recovered as sister to *Anamenia gorgonophila* (bs = 96), the only other member of Strophomeniidae for which molecular data are currently available. Our analysis recovers Proneomeniidae as monophyletic, although with moderate support (bs = 63).

Sequences obtained for the 18S and 28S rRNA markers were relatively short (607 and 1013 bp for *D. gummi* sp. nov. and 593 and 279 bp, *S. boricua* sp. nov. respectively). However, BlastN searches against GenBank showed high similarity to other solenogasters sequences, with matches ranging from 97.55 to 97.64% for 18S rRNA, and from 91.60 to 92.76% for 28S rRNA (Suppl. material 1: table S1).

### ﻿Coral host barcode data

Complete, circular mitogenomes were assembled for two coral hosts with one type specimen. The assembled mitogenome of Sibogagorgia cf. cauliflora mitogenome measured 19,005 bp, whereas the mitogenomes for the two *V. nigrescens* specimens were slightly shorter at 18,730 bp each.

mtMutS barcode sequences from coral hosts were aligned and compared to type specimen sequences. The Sibogagorgia cf. cauliflora host sample was 1.1% (p-distance) divergent from the holotype (USNM 1122229) and one paratype (USNM 1122230) *S. cauliflora* across a 720-bp alignment. Notably, the mtMutS sequence host coral was identical to one paratype (USNM 54831) collected from off northwest of Havana, Cuba. The host *V. nigrescens* sample was 0.1% (p-distance) divergent from the schizotype specimen across the full-length mtMutS sequence (2,958 bp). BlastN searches against the nr/nt collection further confirmed the placement of these two species within the genera *Sibogagorgia* and *Villogorgia* (Suppl. material 1: table S2).

## ﻿Discussion

The two solenogasters described here are the first formally named species from Puerto Rico. Although no species had previously been described from the Puerto Rican region, Linse and Schwabe (2018) reported seven morphospecies from the Puerto Rico Trench. Within the Caribbean, the only other named species was *Neomenia microsolen* Wirén, 1892. Nevertheless, numerous additional specimens from shallow and deep waters around Guadeloupe and Martinique remain undescribed in museum collections (Cobo, unpubl. data). From adjacent areas in the western Atlantic, four species are known off the coasts of Florida and Louisiana: *Proneomenia acuminata* (Wirén, 1892), *Spengelomenia bathybia* (Heath, 1912), *Dondersia tweedtae* Farris, Olson & Kocot, 2024, and *Eleutheromenia bullescens* Cobo, 2024 (Wirén 1892; Heath 1912; Felder and Camp 2009; Cobo et al. 2024b).

Detailed anatomical examination of the two new species revealed key diagnostic characters that support their distinctiveness and clarify their systematic placement. Features of the radula, foregut glands, and reproductive structures proved especially informative for distinguishing species. In *Dorymenia gummi* sp. nov. the exceptionally high number of radular denticles and the absence of seminal receptacles distinguish it from related species. *Strophomenia boricua* sp. nov. is notable for its bundled seminal receptacles, rudimentary radular sac, and foregut gland arrangement. Although external morphology is of limited diagnostic value, features such as sclerite patterns and presence or absence of projections and keels provide helpful clues. These characteristics aid in identification when complemented by anatomical and molecular data.

The two new species display similar external appearance, although closer examination reveals distinctions such as arrangement of sclerites in *Strophomenia boricua* sp. nov. and the body shape in *Dorymenia gummi* sp. nov. SEM studies of the sclerites also reveal differences in sclerite type and in the arrangement of the ventral sclerotome. While these characteristics are informative only at the family level (e.g., Scheltema et al. 2012; Todt 2013; Cobo et al. 2023) they remain essential for preliminary specimen sorting. Live observations and high-quality photographic documentation are particularly valuable for capturing traits that degrade after fixation, such as body coloration. Although these features can occasionally assist species identification (e.g., Nierstrasz and Stork 1940; Salvini-Plawen 1998; Scheltema et al. 2012; Cobo et al. 2024a, b), in this case the habitus alone was insufficient to differentiate the new species from their congeners, reinforcing the necessity of internal anatomical and molecular data for accurate delimitation.

Our phylogenetic analysis confirmed the morphological identification of both new species with *Dorymenia gummi* sp. nov. grouped with other *Dorymenia* species, while *Strophomenia boricua* sp. nov. was recovered as sister to *Anamenia gorgonophila*, providing the first molecular data for the genus *Strophomenia*. In contrast to Cobo et al. (2023), where Proneomeniidae was recovered as paraphyletic in both COI-only and concatenated analyses, our COI tree supports monophyly of the family, although with moderate support (bs = 63). The inclusion of *Strophomenia* in our dataset also alters the topology by recovering an independent clade uniting Strophomeniidae and Epimeniidae (bs= 78). Proneomeniidae clusters with the Strophomeniidae and Epimeniidae clade with a bootstrap support value of 67. Overall while COI provides useful signal for confirming species-level placement, higher-level relationships remain sensitive to taxon sampling. Further multilocus and morphological data, as well as the inclusion of additional taxa (see discussion in Cobo et al. 2023), will be required to clarify the boundaries and evolutionary history of the “Proneomeniidae clade” sensu Cobo et al. (2023) which still exhibits considerable unresolved relationships.

While BlastN searches of the 18S and 28S rRNA sequences revealed high similarity to other solenogasters in GenBank, no matches were found from the same family or genus, limiting their utility. This reflects the historical context in which 18S and 28S have rarely been used for solenogaster barcoding, in contrast to the more widespread use of COI and 16S (Bergmeier et al. 2016, 2017). Similarly, although the use of COI in the context of phylogenetic analysis is reliable, a simple BlastN search is not sufficient for direct species identification, although it is useful for genus or family identification (Suppl. material 1: table S1). This highlights the need to expand the barcode reference library for Solenogastres by incorporating additional genes and a broader representation of taxa.

The fact that both new species were found on octocoral hosts lends support to the hypothesis of specialized ecological relationships between solenogasters and cnidarians (Salvini-Plawen 1972). Given these close associations, we recommend collaboration with coral systematists to accurately identify host species. Our findings further suggest that Solenogastres occupy a variety of hosts spanning multiple octocoral orders, including Scleralcyonacea (*Sibogagorgia*) and Malacalcyonacea (*Villogorgia*). One host, tentatively identified as Sibogagorgia cf. cauliflora, was 1.1% divergent from the holotype and one paratype specimen collected from the Pacific Ocean (Herrera et al. 2010). However, the mtMutS barcode was 100% identical to that of another paratype collected in the Florida Straits off Cuba. As suggested by McFadden et al. (2011), mtMutS genetic distances >0.5% are likely indicative of different species. Therefore, it is possible that the *Sibogagorgia* host represents a new octocoral species in the Caribbean Sea and Florida Keys. Additional morphological and genomic data from collections in both Pacific and Atlantic oceans are needed to confirm species boundaries.

Our observations also highlight the increasing importance and effectiveness of ROVs for discovering and documenting deep-sea biodiversity. Beyond enabling the collection of intact specimens, ROVs provide crucial contextual data on habitat, behavior, and organism-host interactions (Fig. 1). The association of the two new solenogaster species with corals supports the hypothesis that solenogasters use corals as both shelter and food sources. However, further targeted research is needed to determine whether these solenogaster-coral associations exhibit species-specific patterns. ROV footage also provides insights into ecological aspects of Solenogastres biology that remain largely unexplored, including movement patterns, feeding behavior, and reproductive strategies. The use of ROVs has already proven effective in behavioral studies and new species discoveries among other invertebrate groups (e.g., Hunt et al. 2000; Hudson et al. 2004; Caballero-Herrera et al. 2022; Betters et al. 2024; Ekins and Wilson 2024; Giusti et al. 2024). These examples highlight the potential of ROVs to transform our understanding of the biology of marine invertebrates.

The successful inclusion of barcode data from solenogasters and their coral hosts illustrates the effectiveness of genome skimming in integrated taxonomic and ecological studies. Mitogenomes of mollusks can be challenging to assemble from short-read data given that they contain structural rearrangements and repetitive regions and have unusual variations in size (Ghiselli et al. 2021). Despite the challenges of assembling complete mitochondrial genomes, our genome skimming approach successfully yielded partial or full-length DNA barcode sequences that significantly informed our phylogenetic and ecological analyses. Genome skimming of marine invertebrates holds promise for a wide range of applications, including mitogenome and rRNA nuclear operon assembly, as well as recovery of ultraconserved elements (UCEs) and other loci of interest (see Quattrini et al. 2024).

## ﻿Conclusions

This study formally describes two novel solenogaster species *Dorymenia gummi* sp. nov. and *Strophomenia boricua* sp. nov., providing the first records of Solenogastres from waters around Puerto Rico and the Caribbean Sea. Using an integrative taxonomic approach, we document their affinities and host associations. Our findings underscore the continued relevance of anatomical study and the growing value of molecular data in solenogaster taxonomy. Notably, this is the first time sequences have been obtained for the genus *Strophomenia*, contributing to ongoing work to resolve the complex relationships within the ‘Proneomeniidae clade’ sensu Cobo et al. (2023). However, the challenge of characterizing diagnostic internal features through time-consuming histological study remains a significant bottleneck in discovery and description. Our observations contribute to a growing body of evidence that host associations may be useful in species delimitation, but further research is needed. Genome skimming also proved effective for recovering DNA barcodes from both solenogaster specimens and their coral hosts, showing promise for studying ecological interactions. In addition, this work demonstrates the potential of ROV exploration for solenogaster species discovery. Future studies should explore host specificity and broader biogeographic patterns in underexplored deep-sea habitats.

## Supplementary Material

XML Treatment for Dorymenia

XML Treatment for Dorymenia gummi

XML Treatment for Strophomenia

XML Treatment for Strophomenia boricua

## ﻿Additional information

### Use of AI

No use of AI was reported.

### Author ORCIDs

M Carmen Cobo https://orcid.org/0000-0002-8481-2086

Corinna Breusing https://orcid.org/0000-0001-6845-0188

Andrea M. Quattrini https://orcid.org/0000-0002-4247-3055

Santiago Herrera https://orcid.org/0000-0001-7204-9434

Ellen E. Strong https://orcid.org/0000-0001-7181-4114

### Data availability

Newly acquired mtMutS data for the coral hosts and barcodes for the solenogasters have been added to GenBankBioProject = PRJNA1338364. The article is registered in ZooBank (urn:lsid:zoobank.org:pub:B0DE435D-8997-4F12-B664-05273C97A593). Video of the collection event for USNM 1690452 (on USNM 1689348) can be found here: https://www.youtube.com/watch?v=DWOQR23jVH8.

## Supplementary materials

Supplementary material 1 Supplementary tables Data type docx Explanation note **table S1.** BlastN results for COI, 18S, and 28S rRNA sequences of *Dorymenia gummi* and *Strophomenia boricua*. The best match in GenBank is shown for each gene. **table S2.** BlastN results for mtMutS sequences of the coral associates of *Dorymenia gummi* (Sibogagorgia cf. cauliflora) and *Strophomenia boricua* (*Villogorgia nigrescens*). Only the first 600 bp were blasted to avoid biased searches against longer sequences. The best match in GenBank is shown. https://binary.pensoft.net/file/1478273This dataset is made available under the Open Database License (http://opendatacommons.org/licenses/odbl/1.0/). The Open Database License (ODbL) is a license agreement intended to allow users to freely share, modify, and use this Dataset while maintaining this same freedom for others, provided that the original source and author(s) are credited.M Carmen Cobo, Corinna Breusing, Andrea M. Quattrini, Santiago Herrera, Ellen E. Strong
