# Supplementary material for: ﻿First Solenogastres (Mollusca, Aplacophora) from Puerto Rico: descriptions of two new species and notes on their coral hosts
Source: Zookeys. 2025 Nov 26;1261:115–40. doi: 10.3897/zookeys.1261.164889 (PMC12719824; doi:10.3897/zookeys.1261.164889)
Supplement: Supplementary material 1 — Supplementary tables [file zookeys-1261-115_article-164889__-s001.docx]

Supplementary material

**Table S1.** BlastN results for COI, 18S, and 28S rRNA sequences of *Dorymenia gummi* and *Strophomenia boricua*. The best match in GenBank is shown for each gene.

| **Species** | **Gene** | **Acc. no.** | **e-value** | **Bitscore** | **% identity** | **Alignment (bp)** | **Taxonomy** |
| --- | --- | --- | --- | --- | --- | --- | --- |
| *D. gummi* | 18S | FJ649600.1 | 0.0 | 1038 | 97.55 | 611 | *Simrothiella margaritacea* |
|  | COI | OQ597890.1 | 3e-155 | 562 | 82.71 | 637 | *Dorymenia* sp. |
|  | 28S | MG878440.1 | 0.0 | 689 | 91.60 | 512 | *Neomenia carinata* |
| *S. boricua* | 18S | FJ649599.1 | 0.0 | 1020 | 97.64 | 593 | *Wirenia argentea* |
|  | COI | OQ597876.1 | 2e-127 | 470 | 83.67 | 498 | *Anamenia gorgonophila* |
|  | 28S | MG878441.1 | 1.33E-109 | 409 | 92.76 | 290 | *Neomenia carinata* |

**Table S2.** BlastN results for mtMutS sequences of the coral associates of *Dorymenia gummi* (*Sibogagorgia* cf. *cauliflora*) and *Strophomenia boricua* (*Villogorgia nigrescens*). Only the first 600 bp were blasted to avoid biased searches against longer sequences. The best match in GenBank is shown.

| **Species** | **Acc. no.** | **e-value** | **Bitscore** | **% identity** | **Alignment (bp)** | **Taxonomy** |
| --- | --- | --- | --- | --- | --- | --- |
| *S.* cf. *cauliflora* | KC984605.1  PP500889.1 | 0.0 | 1109 | 100.00 | 600 | *Sibogagorgia cauliflora* (Scleralcyonacea; Coralliidae)  *Sibogagorgia* sp. (Scleralcyonacea; Coralliidae) |
| *V. nigrescens* | AY683073.1  HQ694720.2  MT795622.1 | 0.0 | 1109 | 100.00 | 600 | *Villogorgia* sp. (Malacalcyonacea; Plexauridae)  *Muricella abnormalis* (Malacalcyonacea; Anthogorgiidae)  *Muricella* sp. (Malacalcyonacea; Anthogorgiidae) |

**Table S3.** Accession numbers NCBI for the new species (BioProject = PRJNA1338364)

| **Species** | **Gene** | | | **BioSample** |
| --- | --- | --- | --- | --- |
| *D. gummi* sp. nov. | COI | |  |  |
|  | 18S | | |  |
|  | 28S |  | |  |
| *S. boricua* sp. nov. | COI | | |  |
|  | 18S | | |  |
|  | 28S | | |  |
